# High Levels of Straying in Returning, Repeat‐Spawning Atlantic Salmon Between Two Neighbouring Danish Rivers

**DOI:** 10.1002/ece3.73997

**Published:** 2026-07-07

**Authors:** Hugo Flávio, Lene Klubben Sortland, Kim Birnie‐Gauvin, Anders Koed, Søren Larsen, Kim Aarestrup

**Affiliations:** ^1^ National Institute of Aquatic Resources Technical University of Denmark Silkeborg Denmark; ^2^ Department of Biology Dalhousie University Halifax Nova Scotia Canada; ^3^ NINA Norwegian Institute for Nature Research Trondheim Norway; ^4^ Danish Centre for Wild Atlantic Salmon Skjern Denmark

**Keywords:** acoustic telemetry, kelt, marine survival, return migration

## Abstract

Atlantic salmon ( *Salmo salar* ) are iconic for their homing capacity, with most populations migrating into the ocean and eventually returning to their natal river. However, straying is known to occur, with small portions of the population returning to different rivers. Here, we studied the seaward and return migration of Atlantic salmon kelts from wild and hatchery origin, in River Skjern, Denmark. Kelts were captured, tagged and released along River Skjern, or tagged at the local stocking facilities following gamete removal. Of the 199 tagged fish (134 wild and 65 stocked), 110 reached the ocean (55%). Wild‐spawned kelts captured and released along the river were the most likely to survive (63 out of 97, 65.6%) while kelts tagged after stripping and reconditioning at the stocking facilities had the lowest survival rates (5 out of 25, 20%). Only 10 kelts were recorded returning after spending more than 1 year at sea (five wild and five stocked fish). While this constitutes a limited sample size, seven of the 10 returning fish (four wild, three stocked) entered a neighbouring river instead (i.e., strayed). This high straying may be due to (1) a weakening of homing ability with age, (2) the returning fish encountering the neighbouring river first during their southward return‐migration from the Atlantic Ocean and mistakenly entering it given the similarity between the two river systems, (3) some fish actually originating from the neighbouring river, or (4) the influence of long‐term hatchery‐rearing practices, which are known to increase straying rates. Although the small sample size of repeat spawners limits firm conclusions, the observed high straying rates raise concerns for monitoring and management that depend on accurate adult return counts, particularly in systems with a history of stocking.

## Graphical abstract

We studied the seaward and return migration of Atlantic salmon kelts from both wild and hatchery origin in River Skjern, Denmark. Of the 199 tagged animals, 110 reached the ocean (55%). Only 10 kelts were recorded returning after spending more than 1 year at sea.

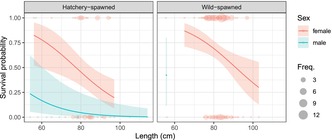

## 1 Introduction

Atlantic salmon ( *Salmo salar* ) are iconic for their homing capacity. As described extensively in literature, most Atlantic salmon are born in freshwater, migrate into the Atlantic ocean, and eventually return to their natal river (Foster and Schom 1989; Jonsson and Jonsson 2011; Klemetsen et al. 2003; McCormick et al. 1998; Potter and Russell 1994). The mechanisms behind this homing ability are not yet completely understood, but there is agreement that fish rely on their olfactory senses to pinpoint their natal river (Foster and Schom 1989; Morin et al. 1989; Morin and Døving 1992; Keefer and Caudill 2014; Stabell 1984). The prevalence of homing behaviour over straying to non‐natal rivers is one of the reasons why Atlantic salmon is managed at the population level (i.e., river basin), in contrast to other non‐homing migratory species. The methods for estimating marine mortality rates for Atlantic salmon also depend on the assumption that the number of returning adults is a proportion of the number of fish that originally left the river. Nevertheless, straying is a natural and relevant component of the behaviour of anadromous salmonids, including Atlantic salmon (Birnie‐Gauvin, Thorstad, and Aarestrup 2019; Jonsson et al. 2003; Keefer and Caudill 2014).

Atlantic salmon are iteroparous; that is, they can reproduce multiple times over the course of their lifetime (Fleming 1996). After a variable growing period at sea (typically 1–3 years), adults return to the freshwater environment to spawn, and some of these may return to the ocean once more. The adults that survive the spawning season are termed kelts (Allan and Ritter 1977). After reconditioning at sea, these fish return to the freshwater environment to repeat the spawning cycle. The majority of repeat spawners tend to be females (Mills 1989; Niemelä et al. 2000), which are usually considerably larger on their second spawning season and can contribute a disproportionately high number of eggs upon repeat spawning (Klemetsen et al. 2003). As such, repeat spawners play an important role in the population's resilience and continuity.

The marine phase of the Atlantic salmon life is particularly challenging to study, especially because the smolts (i.e., juveniles migrating to the sea for the first time) are relatively small. Smolts may be successfully tagged with Passive Integrated Transponder (PIT) tags or acoustic tags, but PIT telemetry has limited range, which limits it use in shore and marine environments, and the acoustic tags deployable on smolts do not last long enough to provide knowledge about their behaviour at sea (battery typically lasts under 60 days). However, out‐migrating kelts offer a window into marine behaviour, as they can accommodate a larger range of tags. Moreover, estimates of Atlantic salmon post‐spawning survival remain scarce (see Halttunen et al. 2013; Jonsson et al. 1990, 1991), but provide important information on the proportion of repeat spawners, and the factors that may promote longer survival. By tagging out‐migrating kelts in River Skjern (Denmark), we aimed to shed more light into their migratory behaviour and survival, both during the seaward and return migrations. Given prior research, we expected: (1) females to survive better than males; (2) males to leave the river earlier than females; (3) individuals in lower condition to out‐migrate earlier; and (4) individuals in lower condition to have lower survival (Bordeleau et al. 2019; Halttunen et al. 2013; Niemelä et al. 2000; Shearer 1992). During the time of the study, acoustic receivers were deployed in a neighbouring river (River Storå) for a different study, and some of the kelts tagged from the present study were (serendipitously) detected in the arrays deployed in River Storå. In the present work, we also explore the consequences of these findings.

## 2 Methods

### 2.1 Study Area

**FIGURE 1:**
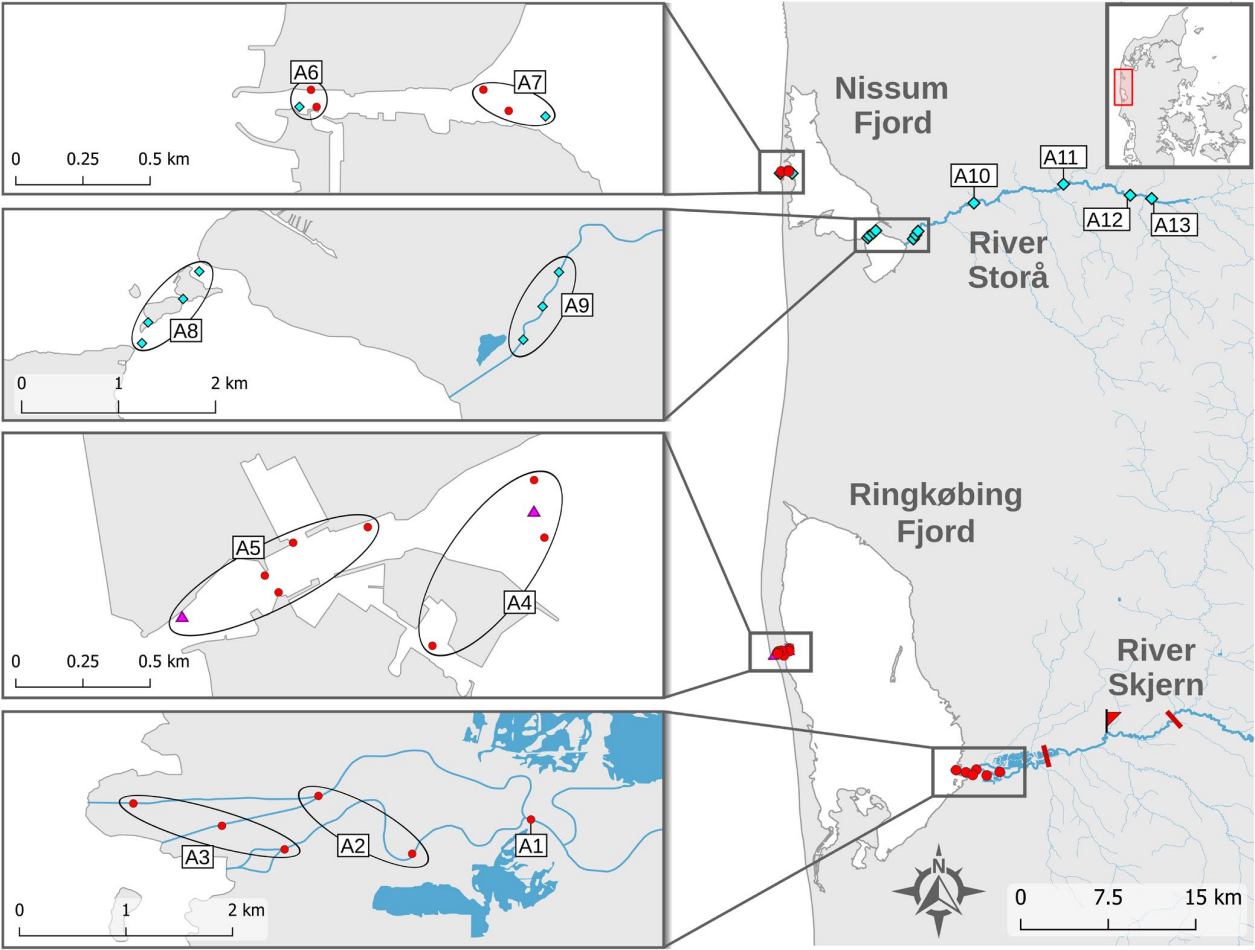
The River Skjern and River Storå systems. Atlantic salmon kelts were tagged and released between the two red bars visible in River Skjern. The red flag represents the Danish Centre for Wild Atlantic Salmon (DCV), where offspring are raised, and where hatchery‐spawned kelts were tagged and subsequently released in the river. Receivers marked with red dots in River Skjern were active the whole study period (2017–2020), and between 2018 and 2020 in River Storå. Receivers marked with cyan diamonds (only present in River Storå) were only active in 2018. Receivers marked with purple triangles (only present in River Skjern) were only active in 2017.

The River Skjern, in Denmark, is well known for its historic Atlantic salmon population (Nielsen et al. 1999). This lowland river has a catchment area of approx. 2500 km^2^, a mean annual flow of 35 m^3^/s, and runs for approximately 95 km before meeting Ringkøbing Fjord and reaching the North Sea (55°55′ N, 8°22′ E; Figure 1). The Atlantic salmon population of the river has been recovering over the recent decades as a result of intensive management. In the early 2000's, after a period of high‐anthropogenic impacts (i.e., draining, damming and channelization), the river was restored to a natural meandering state, multiple barriers were removed, and pollution was controlled as part of a large effort to improve the river's Atlantic salmon population (Koed et al. 2020). Atlantic salmon originating from other European rivers, which had previously been stocked in River Skjern, were systematically removed from the system to restore a wild‐origin population. Part of this process involved extensive supplementary stocking, using a large number of wild‐origin returning adults every year to maintain a broodstock with genetic integrity (i.e., the broodstock was confirmed to have originated from River Skjern). Hatchery‐reared fish are released as juveniles (both as parr and at the smolt stage) after being marked by clipping the adipose fin, thus enabling detection upon recapture. Adipose clipping is a common practice for stocked fish as it is a reliable method to distinguish between wild and hatchery fish, allowing practitioners to later assess whether stocking is having a beneficial impact on the population. The Atlantic salmon population in River Skjern is therefore supported by the spawning of both wild and stocked origin adults, and the stocking of juveniles every year.

River Storå is situated northward of River Skjern and has a growing Atlantic salmon population. As the result of integrative management plans, the Atlantic salmon population of River Storå is now self‐sustaining and, as such, the release of hatchery‐reared individuals in this river was discontinued in 2017 (Koed et al. 2020). However, monitoring efforts in this river have repeatedly revealed the presence of fish whose adipose fin had been clipped, which fuelled a suspicion that at least part of the adults returning to River Storå actually originated from stocking in River Skjern, where all stocked Atlantic salmon have their adipose fin clipped (Koed et al. 2020). River Storå has a catchment of 825 km^2^, a mean annual flow of 10 m^3^/s, and runs for approximately 104 km before entering Nissum Fjord and reaching the North Sea. This system is very much alike to River Skjern, with both catchments being lowland and having similar geomorphological and hydrological characteristics.

In both river systems, one‐sea‐winter Atlantic salmon tend to return in late summer and autumn, while multi‐sea‐winter fish return in the spring and early summer (local observations). From these, the proportion of repeat‐spawners is unknown in River Skjern, and scale readings from River Storå indicate circa 8% of the returns are repeat‐spawners (Lindvig 2011).

### 2.2 Experimental Fish

In this study, Atlantic salmon kelts of both stocked origin and wild origin were used. Additionally, kelts were either tagged after stripping and spawning at the hatchery (Danish Centre for Atlantic Salmon, DCV) or directly along River Skjern, after spawning in the wild. As such, the tagged kelts can be discriminated into four groups based on their origin and capture location: (1) hatchery‐spawned kelts of stocked origin (stocked‐DCV), (2) wild‐spawned kelts of stocked origin (stocked‐river), (3) hatchery‐spawned kelts of wild origin (wild‐DCV) and (4) wild‐spawned kelts of wild origin (wild‐river). All fish tagged at DCV were captured shortly before the spawning season (in December), and their gametes used for the broodstock. Hatchery‐spawned kelts therefore spent ~2 months at the stocking facilities between their capture and release (more details below). A subset of the data used in the present study (i.e., the wild kelts captured along River Skjern in 2017) were used in a separate study by Birnie‐Gauvin, Flávio, et al. (2019), which aimed to disentangle the effects of cortisol on migration timing and success for both Atlantic salmon and anadromous brown trout ( *Salmo trutta* ) kelts.

#### 2.2.1 Kelts Tagged at DCV

Every year, in the first 2 weeks of December, DCV captures wild and hatchery‐reared upstream‐migrating Atlantic salmon along River Skjern using electrofishing (Stampes Elektro, SE 500, Ringkøbing, Denmark). These fish are then transported to DCV in ~6500 L tanks. Broodstock adults are kept in captivity and stripped, also during the month of December. Once stripped of eggs and sperm, the kelts used in this study were returned to the holding tanks and allowed to recover until the tagging period. In total, hatchery‐spawned kelts tagged at DCV spent approximately 2 months (December and January) at the stocking facilities before being released. Once tagged, the kelts were transported and released in the nearest river point (see the red flag in Figure 1).

In total, 9 stocked‐ and 15 wild‐origin hatchery‐spawned kelts were tagged in 2017, and 16 stocked‐ and 18 wild‐origin hatchery‐spawned kelts were tagged in 2018. Detailed numbers of tagged fish, as well as biometric information, are provided in Table 1.

**TABLE 1:** Summary information of the tagged Atlantic salmon kelts.

Year	Origin	Capture	*n* (males)	Length (cm)	Mass (kg)	Condition (K)
2017	Stocked	DCV	9 (3)	78.0 (15.4)	3.4 (1.8)	0.66 (0.04)
	Stocked	River	24 (0)	81.0 (4.7)	3.5 (0.7)	0.64 (0.06)
	Wild	DCV	15 (1)	81.0 (8.6)	3.7 (1.3)	0.66 (0.10)
	Wild	River	44 (1)	81.2 (6.6)	3.6 (0.8)	0.66 (0.07)
2018	Stocked	DCV	16 (3)	82.8 (10.0)	4.0 (1.6)	0.67 (0.05)
	Stocked	River	16 (0)	82.6 (6.6)	3.8 (1.4)	0.66 (0.08)
	Wild	DCV	22 (4)	81.9 (11.2)	3.9 (2.0)	0.66 (0.07)
	Wild	River	53 (1)	83.4 (6.9)	3.8 (0.9)	0.65 (0.05)

*Note:* The data in the biometric columns (length, mass and condition) are presented as the mean with the standard deviation in parentheses. The ‘*n*’ column shows the total number of fish tagged, and the corresponding number of males in parentheses. Hatchery‐spawned kelts that were tagged and released at the Danish Centre for Atlantic salmon are labelled as ‘DCV’ in the Capture column.

#### 2.2.2 Kelts Tagged in the River

Wild and hatchery‐reared Atlantic salmon kelts were captured for tagging using electrofishing (Stampes Elektro, SE 500, Ringkøbing, Denmark) along River Skjern between the 17^th^ of January and the 6^th^ of February 2017, and between the 18^th^ and 24^th^ of January 2018. Electrofishing did not result in bruising or any other visible damage to the fish. All kelts captured were immediately placed in a 500 L container with fresh, oxygenated, stream water. Kelts tagged in the river were immediately processed (i.e., undergone sampling, tagging surgery and released after recovery). The tagging period was selected to ensure the typical spawning period in Denmark had passed (Christensen et al. 1993). In addition, the condition factor and the presence of scar tissue from spawning activity were used to confirm that the tagged fish were indeed kelts and not late spawners (Jonsson et al. 1997).

In total, 24 stocked and 44 wild kelts were tagged along the river in 2017, and 16 stocked and 52 wild kelts were tagged along the river in 2018. Detailed numbers of tagged fish, as well as biometric information, are provided in Table 1. The sex determination revealed that most of the tagged kelts were female (186/199). This is expected, as the majority of the repeat‐spawners are typically females and males may return to sea earlier than females (Halttunen et al. 2013; Mills 1989; Niemelä et al. 2000; Shearer 1992).

### 2.3 Tagging Procedure

Fish were anaesthetized in a solution of 0.03 g/L benzocaine in freshwater until their ventilation rate had slowed significantly. The fish were measured, weighed and tagged with an acoustic tag through a 2 cm incision on the left lower side of the fish, anterior to the pelvic fins. The acoustic tags used were ThelmaBiotel ID‐LP13 tags (13 mm diameter, 28 mm length), weighing 9.2 g in air and 5.5 g in water, with a transmission rate of 30–90 s and an expected battery life of 2 years. Each incision was closed with two 4–0 vicryl absorbable sutures. Surgical implantation was performed by an experienced fish surgeon in compliance with local regulations. Additionally, a scale sample and a small fin sample were also obtained from each individual for genetic determination of the fish's sex. Fish were then left to recover in a 200 L container of fresh river water for 5–10 min until fully responsive to touch and released at the site of capture (for fish captured along River Skjern), or at the closest river point from DCV (for fish tagged at DCV). All kelts were fully responsive at the time of release, with no noticeable injuries and no residual eggs. Very small amounts of fungus were present on a few individuals but not enough to raise concerns. All handling and tagging procedures are in accordance with the Danish Experimental Animal Inspectorate (licence number 2017‐15‐0201‐01164).

### 2.4 Sex Determination

DNA was extracted from 3 to 4 scales using 50 μL Chelex 10%, 10 μL Proteinase K. All PCR amplifications were performed in a final volume of 6 μL with 0.5 μL template DNA and a concentration of 0.2 μM for each primer. PCR reactions were conducted following the kit manufacturer's recommendation, using an annealing temperature of 57°C and 30 cycles. The PCR product was run on a Seq Studio Automated Sequencer, with Genemapper (version 4) for genotyping. Sexing was done using the sdY primer (Quéméré et al. 2014) with a peak presence at 180–200 base pairs corresponding to males and the absence of a peak corresponding to females. Two other primers (ssa85 and SsOsl408) were used for positive control.

### 2.5 Data Analysis

#### 2.5.1 Acoustic Data Validity

Raw acoustic data were checked for potentially flawed detections using the R package actel 1.4.0 (Flávio and Baktoft 2021). Events flagged as potentially flawed (e.g., going through several arrays undetected) were investigated in detail so that any erroneous detections could be identified and removed. Receiver detection efficiency during the out‐migration period was calculated through analytical Cormack–Jolly–Seber modelling (Perry et al. 2012), and efficiency for array A5 was estimated by comparing tags detected at the two eastern receivers with tags detected at the three/two western receivers (in 2017 and 2018, respectively), using the same R package (see also section ‘Using replicated telemetry arrays to estimate detection probability’ in Perry et al. 2012). Array efficiency during seaward migration remained above 90% both in 2017 and 2018, with the exception of array A4 in 2018, for which efficiency was estimated to be 71% (Table 2). Intra‐array efficiency estimates at A5 showed 98.9% and 99.7% efficiency in 2017 and 2018, respectively. The high estimated detection efficiency at the last array (with 100% within the credible range; Table 2) and the fact that all kelts that were detected returning from the ocean had been detected leaving Ringkøbing Fjord gives high confidence that all successful seaward migrants were detected leaving the Fjord.

**TABLE 2:** Estimated detection efficiencies of acoustic arrays (A1–A5) deployed in River Skjern and Ringkøbing Fjord, 2017 and 2018.

Year	A1	A2	A3	A4	A5
2017	90.7 (83.2–95.7)	96.8 (91.5–99.2)	100 (100–100)	71.4 (58.3–82.5)	98.8 (92.3–100)
2018	86.1 (78.1–92.2)	93.7 (87.5–97.5)	100 (100–100)	92.1 (83.5–97.2)	99.7 (98.1–100)

*Note:* Estimates are shown as percentages (%), with corresponding 95% confidence intervals in brackets.

#### 2.5.2 Seaward Survival

A Bernoulli generalised linear model (GLM) with logit link function (i.e., a logistic regression) was applied to test for effects of capture location (categorical, two levels: river or DCV), origin (categorical, two levels: wild or stocked), length (continuous), mass (continuous), sex (categorical, two levels: female or male) and year (categorical, two levels: 2017 or 2018) on seaward survival probability, measured as detection on any of the receivers that composed array A5. The model was checked for overdispersion by fitting a quasibinomial distribution (logit link). As the dispersion parameter was estimated to be 1.02, the model was returned to a Bernoulli distribution. Stepwise chi‐square goodness‐of‐fit model selection was performed to determine which covariate combination would produce the best model. The initial model equation was as follows:

$$\begin{array}{c} {\text{Survived}}_{i}\sim \text{Bernoulli}\left(\mu_{i}\right)\\ E\left(\text{Survived}\right)=\mu_{i}\\ \text{logit}\left(\mu_{i}\right)={\text{Capture}}_{i}+{\text{Origin}}_{i}+{\text{Length}}_{i}+{\text{Condition}}_{i}+{\mathrm{Sex}}_{i}+{\text{Year}}_{i} \end{array}$$

#### 2.5.3 Return Migration

Only a small number of kelts were detected returning from the ocean (10 out of 199 tagged). As such, no additional statistical analyses were performed to attempt to find patterns between successful and unsuccessful returns. Nevertheless, the behaviour of the returning kelts is examined in detail in the results.

## 3 Results

### 3.1 Seaward Survival and Behaviour

**FIGURE 2:**
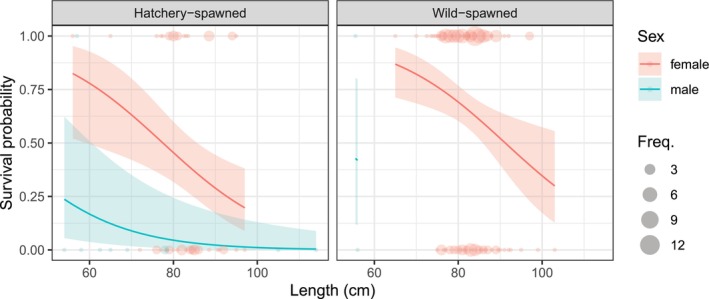
Factors affecting seaward survival of Atlantic salmon kelts in River Skjern. Fish tagged at the Atlantic salmon rearing facilities (DCV) were less likely to reach the ocean. Females were more likely to reach the ocean than males, and smaller kelts were more likely to survive. Fish of both origins (wild and stocked) are grouped in each panel.

Of the 199 kelts tagged, 160 entered Ringkøbing fjord (80%) and 110 (55% of the total) successfully reached the ocean (52 in 2017 and 58 in 2018). The GLM revealed that origin (wild vs. stocked) did not impact seaward survival (GLM: χ^2^ = 2.1, *p* = 0.15). However, wild‐spawned kelts captured and released along the river were 1.3–1.8 times more likely to survive than hatchery‐spawned kelts tagged at the stocking facilities (reference values provided for females between 70 and 90 cm; GLM: $\chi^{2}$ = 8.648, *p* = 0.003; Figure 2); smaller kelts were more likely to survive (average 1.5% survival chance drop per cm increase for wild‐spawned females; GLM: $\chi^{2}$ = 8.898, *p* = 0.003; Figure 2); and female kelts were also 7–12 times more likely to survive than males (reference values provided for hatchery‐spawned kelts between 70 and 90 cm, where the majority of males was tagged; GLM: $\chi^{2}$ = 10.431, *p* = 0.001; Figure 2). It is important to note that although male kelts were generally smaller than females (${\bar{L}}_{♂}$ = 71.6 cm, ${\bar{L}}_{♀}$ = 82.7 cm), the range of lengths for both sexes was similar (56–103 cm for females, and 54–114 cm for males). It is also important to reinforce that most male kelts were hatchery‐spawned and tagged at DCV, so there could be an overlapping effect between the two variables. However, exploratory variance inflation factor (VIF) tests did not reveal sex and capture site to be prohibitively correlated (i.e., VIF under 1.5 for all variables used in the final model).

**FIGURE 3:**
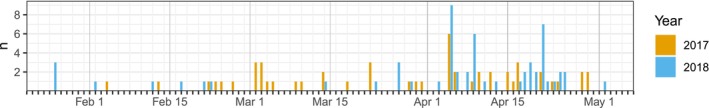
Departure day of year for the kelts detected leaving Ringkøbing Fjord (i.e., last detection at array A5).

**FIGURE 4:**
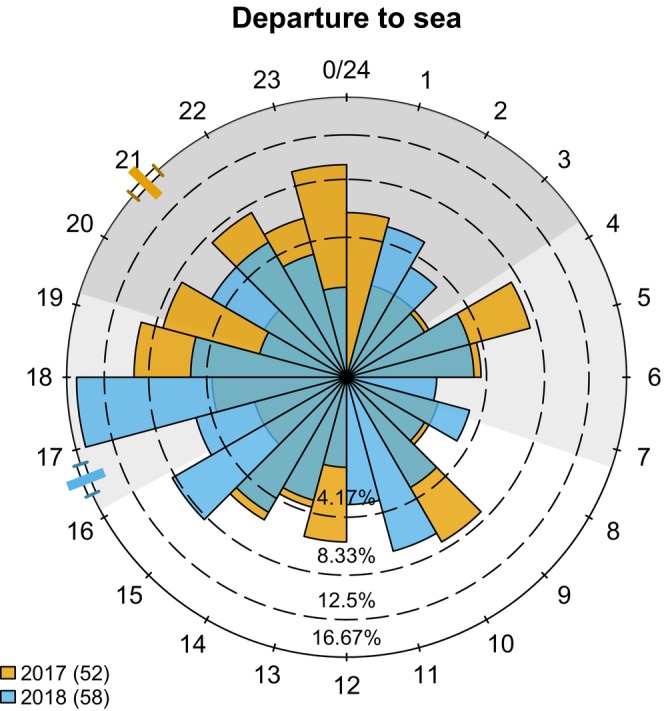
Departure time of day for the Atlantic salmon kelts detected leaving Ringkøbing Fjord (i.e., last detection at array A5). The coloured markers in the outer circle indicate the mean value for the respectively coloured year. The ranges around each of the mean values show the SEM. The number of kelts recorded for each year is presented between parentheses in the respective caption. Each year's bars sum to 100%. The sunrise and sunset times vary greatly over the season; the night time (between sunset and sunrise) in early February is shown by the light grey shaded area, while the night time in late April is shown by the dark grey area.

The successful kelts arrived at sea between late January and early May, with an increase in the number of ocean arrivals during April (Figure 3). Overall, the departing kelts showed a tendency to enter the ocean in the late afternoon (Figure 4). Date of departure was significantly related to the date of release, with every passing day in release leading to a delay of approximately 2 days in departure date (GLM: $\chi^{2}$ = 4.162, *p* = 0.041).

### 3.2 Return Survival and Migration

**FIGURE 5:**
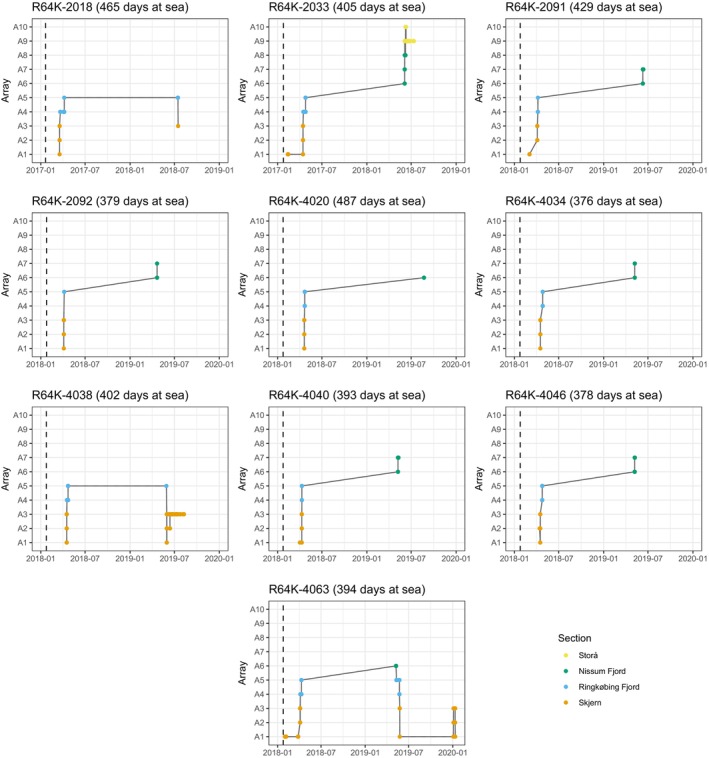
Movements of Atlantic salmon kelts detected returning from the sea. The dashed vertical lines indicate the time of release. Arrays A8–A10 (in River Storå) were only active in 2018. As such, for most of the kelts that returned to Nissum Fjord, we do not know if they continued into River Storå. Kelt R64K‐4063 was detected in Nissum Fjord before returning to Ringkøbing fjord. It was also the only kelt potentially detected after a new spawning event in January 2020, at the limit of the battery life for the tag. For a correspondence between array names and their respective locations, please refer to the study area map (Figure 1).

From the 110 kelts that were detected leaving Ringkøbing Fjord, 10 were detected returning from the ocean (two from 2017 and eight from 2018). All of the returning kelts were wild‐spawned females who had been captured and released along River Skjern (Table 3). Of these 10, eight returned to Nissum Fjord (the entrance to the River Storå basin), rather than their home basin, and only one of those eight (R64K‐4063; Figure 5) eventually turned around, left Nissum Fjord and entered Ringkøbing Fjord. Of the seven fish that strayed and did not turn around, one (R64K‐2033; Figure 5) was detected entering River Storå because its return coincided with a smolt migration study performed in 2018, and another five were detected on the inner side of the Fjord canal (array A7) in 2019. Of the three fish that ultimately returned to Ringkøbing Fjord, all were detected at the mouth of River Skjern, and one (R64K‐4063; Figure 5) was detected again in January 2020 at the limit of the battery life of the tag, potentially returning from another round of successful spawning. All returning fish spent more than 1 year at sea (range: 376–487 days), revealing an alternate spawning pattern. Fish returned between April and July, though most individuals returned in May. Individual departure and return dates, as well as the corresponding total days spent at sea for the returning fish, can be found in Table 3.

**TABLE 3:** Summary information on the migration of the repeat spawning *Atlantic salmon* detected returning from the ocean.

Signal	Year	Origin	Length (cm)	Last seaward detection	First return detection	Days at sea	Strayed
2018	2017	Stocked	83	2017‐04‐06 18:24:29	2018‐07‐15 12:03:33	465	No
2033	2017	Stocked	75.5	2017‐04‐25 07:23:16	2018‐06‐04 15:09:43	405	Yes^a^
2091	2018	Stocked	79	2018‐04‐06 22:18:35	2019‐06‐10 03:43:39	429	Yes
2092	2018	Wild	80	2018‐04‐06 15:13:36	2019‐04‐21 10:16:44	379	Yes
4020	2018	Wild	84	2018‐04‐22 01:57:34	2019‐08‐22 12:40:12	487	Yes
4034	2018	Wild	82	2018‐04‐26 18:00:40	2019‐05‐08 02:56:45	376	Yes
4038	2018	Stocked	85	2018‐04‐22 10:31:53	2019‐05‐29 11:41:04	402	No
4040	2018	Stocked	74	2018‐04‐10 04:38:18	2019‐05‐08 04:12:24	393	Yes
4046	2018	Wild	83	2018‐04‐24 16:25:30	2019‐05‐07 15:53:10	378	Yes
4063	2018	Wild	86	2018‐04‐10 04:21:30	2019‐05‐09 12:21:33	394	No^b^

*Note:* All returning fish were wild‐spawned females, that is, tagged and released along the River Skjern (as opposed to hatchery‐spawned kelts—tagged and released at the Danish Centre for Atlantic Salmon).

^a^ Kelt 2033 returned to Nissum Fjord while a parallel study was being performed. This allowed the detection of the returning kelt all the way to arrays A9 and A10 in River Storå.

^b^ Kelt 4063 was detected at A6 (Nissum Fjord) but then moved into Ringkøbing Fjord and River Skjern, with the first detection at A5 on 2019‐05‐11 05:59:46.

## 4 Discussion

### 4.1 Seaward Migration

More than half (55%) of the tracked Atlantic salmon kelts were detected leaving Ringkøbing Fjord and entering the sea. This contrasts with the 92% survival found by Halttunen et al. (2009) for the first 30 km of the Alta Fjord, in Norway, and the 90% survival reported by Hubley et al. (2008) for River LaHave, in Nova Scotia. The lower survival observed in this study may be explained by: (1) a predation hazard, driven by the presence of seals ( *Phoca vitulina* ), cormorants (*Kalacrocorax carbo*), otters ( *Lutra lutra* ) and possibly the invasive American mink (*Neovision vision*), (2) a by‐catch hazard, driven by the common whitefish ( *Coregonus lavaretus* ) gill‐netting activities that take place in Ringkøbing fjord (where salmonid fisheries are not allowed) and (3) the shape of Ringkøbing Fjord, which is technically a lagoon whose narrow exit to sea is regulated by sluice gates. The fjord's shape likely increases the time and effort the kelts must expend to reach the open sea, spending more time in an area with a high density of predators as well as fisheries activities.

The analyses revealed a significant lower survival chance for hatchery‐spawned kelts that had been held at the local Atlantic salmon stocking facility (DCV). However, most males were tagged at DCV, and males are often less likely to survive spawning events than females, largely due to the high energetic costs of developing secondary sexual traits and males competing aggressively for mates (Mills 1989; Niemelä et al. 2000; Shearer 1992). In this study, holding the fish in the hatchery for 1–2 months before release likely added additional stress and energetic costs on top of those from reproduction, particularly for males. Consistent with this, Bordeleau et al. (2018) demonstrated that hatchery‐spawned Atlantic salmon kelts exhibited earlier seaward migration (by ~66 days), lower survival to sea, and a 0% repeat spawning rate compared to 6.5% in wild fish. It should be noted, however, that very few males were captured in the river in this study (*n* = 2), likely because males left before females (Halttunen et al. 2013) and have higher mortality rates during spawning. As a result, the higher proportion of males among hatchery‐spawned kelts could have inflated our estimate of hatchery mortality compared to wild‐spawned kelts. Nevertheless, the low survival of hatchery‐spawned kelts is concerning, as collecting wild fish for broodstock remains a widely used management practice for Atlantic salmon. Future studies should investigate how different hatchery holding durations and procedures impact the post‐release survival of kelts, potentially drawing from existing expertise in reconditioning steelhead kelts ( *Oncorhynchus mykiss* ; e.g., Penney and Moffitt 2014; Penney et al. 2016), to better guide broodstock operations and minimize impacts on wild populations.

Smaller kelts were generally more likely to survive the seaward journey than larger kelts. This could represent a difference between younger and older fish (i.e., first time spawners and repeat spawners), as older fish tend to be larger. Given that reproductive investment is generally size‐dependent, such that energetic depletion increases with size during reproduction (Berg et al. 1998; Jonsson et al. 1997; Klemetsen et al. 2003), larger fish in the present study likely spent more resources on reproduction than smaller fish. In turn, this may have compromised the post‐spawning survival of larger individuals, as insufficient resources may have remained to complete the seaward migration and successfully reach oceanic feeding grounds (Rikardsen et al. 2021). Reproduction is the most energetically‐demanding life stage in many organisms, and is at the centre of one of the most important life history trade‐offs: the partitioning of limited resources into current reproduction versus survival and/or future reproduction (Stearns 1992). The trade‐off between current versus future reproduction is particularly striking in capital breeders, such as Atlantic salmon, which depend on energetic resources accumulated prior to breeding to migrate to spawning grounds, produce gonads, compete, spawn and subsequently return to sea (Jager et al. 2008; Stearns 1992; Stephens et al. 2009). Within this context, larger fish likely opted to invest more in current reproduction at the cost of their subsequent survival, while smaller fish likely opted to increase their odds of survival at the cost of lower current reproductive output.

### 4.2 Return Migration

Returning kelts entered the fjord systems between April and July, 5–8 months in advance of the spawning season, which corresponds well with the normal season for returning 2‐4 SW Atlantic salmon in the region. To do so requires significant endogenous energy reserves given that Atlantic salmon do not feed in freshwater prior to spawning. Furthermore, all returning kelts revealed an alternate‐spawning strategy, spending more than one full year at sea before returning (i.e., skipped one spawning season). This is in line with the behaviour described for populations in Norway (e.g., Niemelä et al. 2006), and contrasts with the more common consecutive‐spawning strategy reported for populations on the western shore of the Atlantic Ocean (e.g., Reddin et al. 2011). This finding has important implications for fecundity, as alternate repeat spawners tend to have larger eggs with better survival rates than consecutive repeat spawners, despite having a lower absolute number of eggs (Reid and Chaput 2012).

In the current study we found a high straying rate into Nissum Fjord and likely River Storå (7 out of the 10 returning fish), with an additional fish entering Nissum Fjord but eventually turning back and being detected at Ringkøbing Fjord. This is considerably higher than the straying rates previously reported for Atlantic salmon. For example, Ulvan et al. (2018) found a straying rate of 9% for maiden Atlantic salmon originating from the River Altaelva, Norway; Keefer and Caudill (2014) found an average 10.1% straying rate for Atlantic salmon populations; and B. Jonsson et al. (2003) reported straying rates of 5.8% for wild and 15.4% for hatchery‐origin Atlantic salmon returning to River Imsa, in Norway. Several factors may explain the high straying of repeat spawners in this study: (1) a weakening of homing ability with age (Keefer and Caudill 2014), (2) a likely southward return‐migration from the ocean bringing migrants past Nissum Fjord before Ringkøbing Fjord, increasing the chance of mistaking the Storå basin for Skjern's, (3) some wild fish caught in Skjern could themselves be strayers originating from River Storå, and (4) the influence of hatchery‐rearing, as wild individuals that descend from stocked parents may stray more than they would naturally (Jonsson et al. 2003; Keefer and Caudill 2014), and both river Storå and Skjern have a long history of stocking and gene flow between them (Nielsen et al. 1999). The very small sample size of repeat spawners (*n* = 10) limits the strength of our conclusions, and future studies should explore straying rates in repeat and maiden spawners using larger sample sizes of wild and hatchery‐reared fish. Still, the high straying of repeat spawners observed in this study raises concerns, as straying could bias adult return counts used in population monitoring and estimates of marine survival for both donor and recipient populations (Miller et al. 2012; Flávio et al. 2020; Sortland et al. 2025). As many Atlantic salmon populations have a history of stocking, future research should examine the extent to which hatchery practices have shaped straying behaviour across systems.

### 4.3 Methodological Considerations

When interpreting the results from this study, it is important to consider that telemetry methods have the inherent risk of recording a behaviour that is no longer representative of the tagged fish. For example, a predator eating a tagged fish and moving with the tag in its stomach can lead to flawed detections (Gibson et al. 2015; Klinard et al. 2019). However, considering the size of the tagged Atlantic salmon, we find it unlikely that an animal would easily consume the entire fish and consequently ingest the tag too. Seals, which may be large enough to consume the entire fish, typically only consume the guts. Nevertheless, because the tagged fish are expected to move towards the ocean, tag ingestion by a predator would be recognisable if it led to unexpected behaviour. The preliminary data analysis did not reveal any movements that could be indicative of predation.

Alternatively, the acoustic receiver arrays may fail to detect a passing tag. Should this happen, the respective fish would erroneously be classified as having died in the study area, when in fact it migrated out. However, array A5 showed high efficiency rates in both years, and all the kelts detected returning from the ocean had also been detected leaving Ringkøbing Fjord. Together, these two facts indicate a very low chance that some kelts may have left Ringkøbing Fjord undetected.

Lastly, since the acoustic transmitters used in this study had an expected lifetime of 2 years, some of the kelts could have returned after two or more years without being detected. A follow up study with longer lasting transmitters and/or the use of passive tagging could shed more light on whether there are repeat spawners that skip more than one spawning season at sea.

## 5 Conclusion

Our study showed that 55% of the tagged Atlantic salmon kelts were able to leave the fjord and return to the ocean, with the peak in departures occurring in April. Kelts captured in the wild were 30%–80% more likely to survive the seaward journey than those that had been stripped for the local broodstock program, but interestingly the origin of the animal (born in captivity vs. born in the wild) had no impact on seaward survival chance. All the animals had departed by May of their respective release years and, 2 years after their departure, 10 kelts (5% of the total tagged, 9% of the total that reached the ocean) were recorded returning for another spawning season. While this consists of a limited sample size, our findings clearly indicate that an unexpectedly large proportion of repeat spawners leaving River Skjern may be returning to a non‐natal river upon their subsequent return to freshwater. The long history of stocking may have elevated straying rates in River Skjern and its neighbouring River Storå. Although the small sample size limits firm conclusions, potentially high straying rates in repeat spawners have important implications for the management of this population and raise questions regarding the extent of straying in first time spawners. Moreover, the present results indicate very low survival of hatchery‐spawned kelts upon their seaward migration and 0% repeat spawning in those individuals, suggesting that hatchery‐spawning has severe negative consequences for kelt survival. Since repeat spawners play an important role in population stability, improving our understanding of the behaviour and survival of this understudied life‐stage is crucial to effectively conserve and manage Atlantic salmon populations.

## Author Contributions

**Hugo Flávio:** data curation (lead), formal analysis (lead), validation (lead), visualization (lead), writing – original draft (lead), writing – review and editing (lead). **Lene Klubben Sortland:** formal analysis (supporting), validation (supporting), writing – original draft (supporting), writing – review and editing (equal). **Kim Birnie‐Gauvin:** investigation (lead), project administration (equal), writing – review and editing (equal). **Anders Koed:** conceptualization (equal), funding acquisition (equal), methodology (equal), writing – review and editing (equal). **Søren Larsen:** investigation (equal), project administration (equal), writing – review and editing (equal). **Kim Aarestrup:** conceptualization (equal), funding acquisition (equal), investigation (equal), project administration (equal), writing – review and editing (equal).

## Funding

This work was supported by the European Maritime and Fisheries Fund (SMOLTRACK III), Danish Net and Fish License, and Innovation Fund Denmark.

## Conflicts of Interest

The authors declare no conflicts of interest.

## Data Availability

The data, scripts and actel‐generated reports used to conduct the analyses reported in this paper are available online at http://doi.org/10.5281/zenodo.7212446.
